# Same but different? A qualitative analysis of the influence of COVID-19 on law enforcement and organized crime in Germany

**DOI:** 10.1007/s12117-022-09470-1

**Published:** 2022-11-14

**Authors:** Sarah Schreier, Katharina Leimbach

**Affiliations:** 1 Institute of Criminology, Eberhard Karls Universität Tübingen, Sand 7, 72076 Tübingen, Germany; 2 Institute for Interdisciplinary Research on Conflict and Violence, Universität Bielefeld, Universitätsstraße 25, 33615 Bielefeld, Germany

**Keywords:** Organized crime, Germany, Law enforcement personnel, Qualitative interviews, COVID-19, Reflexive criminology, Grounded Theory, Situational Analysis

## Abstract

Criminological research on COVID-19 and its repercussions on crimes, criminals and law enforcement agencies is still in its infancy. This paper fills that void with regard to the influence of COVID-19 on organized crime and the work of law enforcement agencies’ investigations of organized crime in Germany by presenting empirical findings from a nationwide qualitative interview study. Through the methodological combination of Grounded Theory and Situational Analysis, we find three central narratives (us vs. them, nationalization vs. internationalization, conservatism vs. innovation) that were provided by law enforcement personnel in terms of the way in which COVID-19 influenced both organized crime groups and their work in the investigation thereof. Following a reflexive approach, the implications of COVID-19 on the research process itself are also discussed.

## Introduction

In November of 2019, when reports about the first cases of the newly discovered “Coronavirus” – SARS-CoV-2 – came out, no one would have thought that this virus would completely change life as we knew it. Considering the severe disruption caused by COVID-19, it is safe to assume that the pandemic with “stay-at-home-orders”, school closings and nationwide lockdowns – in some way or other – influenced almost all areas of life including crime. According to Campedelli et al (2020) such drastic decreases in mobility, at least in the Los Angeles area, led to a decline in crimes such as pick-pocketing or robberies (Campedelli et al 2020). Similarly, Nivette et al (2021) found a substantial drop of an average -37% in urban crime in their study on 27 cities worldwide due to stay-at-home mandates (Nivette et al 2021: 873). While Bullock and Pellegrino (2021) based on data from Rio de Janeiro found similar decreases in extortion and property crimes due to stay-at-home mandates and mobility restrictions, they did not find a decrease in violent crimes. They argue that this has to do with the fact that lethal violence, for the most part, is less random and not dependent on “people being ‘in the streets’” (Bullock and Pellegrino 2021: 161). Supposedly, organized crime as one of the major types of serious crime was also influenced by the pandemic. According to the national statistic on organized crime, organized crime groups’ criminal activities known to German law enforcement agencies primarily include drug-related crimes (e.g. drug trafficking, dealing drugs), cases of fraud, theft and human trafficking (Bundeskriminalamt 2021). Within the German context, organized crime is defined as a type of crime being committed by more than two individuals working together over an extended period of time who are driven by the strive for profits and/or power utilizing either business-like structures, aggression or violent acts of intimidation, or bribes and/or threats to influence and infiltrate public institutions e.g. politics, media, judiciary or the economy (Bundeskriminalamt 2021). While this definition of organized crime is employed by both German law enforcement agencies and the courts since the 1990s, data from our major interview study with law enforcement personnel, on which this paper is based, suggests that 30 + years later organized crime still appears to be a difficult phenomenon to tackle as the concept of organized crime in practice appears to be much more vague and fluctuating than the official definition suggests.

Since the majority of empirical data on organized crime in Germany dates back to the late twentieth century and the beginning of the twenty-first century, the German Federal Ministry of Education and Research currently funds an interdisciplinary research alliance “Organized Crime 3.0”.^1^ The research alliance was launched in late 2020 in order to generate new empirical knowledge on organized crime in Germany. Within this alliance, our study is comprised of a wide variety of different types of data (e.g. official crime statistics, parliamentary documents, judicial rulings, criminal case files, data from an online survey, interview transcripts) and combines both quantitative and qualitative methodological approaches into a mixed-methods research design with the goal to generate new empirical insights as to the big question: What is organized crime and how is it both interpreted and dealt with by German law enforcement agencies? As the research alliance took up work in the midst of the first year of the pandemic, it is undeniable that this state of worldwide emergency also impacted and continues to impact the way that research – especially our qualitative interview research – is conducted. As such, in addition to presenting our empirical findings on the nexus of organized crime in Germany and COVID-19, we also discuss several COVID-19-related obstacles to conducting qualitative research that we experienced first-hand in our study.

Starting off with a literature review on organized crime in general, the nexus of organized crime and law enforcement as well as organized crime and COVID-19, we then situate our research within our methodological framework. This is followed by the presentation of empirical findings from our ongoing nationwide interview study with members of law enforcement agencies regarding our research question: Which narratives do law enforcement agencies provide for the impact of COVID-19 on organized crime and their work in the investigation thereof? Our analysis of our data reveals three central narratives (us vs. them, nationalization vs. internationalization, conservatism vs. innovation) as to the way in which COVID-19 influenced both organized crime groups and the work of law enforcement agencies. Additionally, in line with a reflexive research approach, we also consider the way in which our qualitative research practices were influenced in pandemic times.

## Literature review – what do we know about organized crime, law enforcement and COVID-19?

While organized crime groups in some form or other have fairly certainly existed since the beginning of times, it is only within the last century that organized crime has been made the topic of empirical research. As the phenomenon itself and its definition can vary in different international legal systems thereby making it difficult to equivalate all research on organized crime, this literature review centers around general empirical findings on organized crime in Germany and beyond rather than focusing on inherent definitional discrepancies on the international level.

According to a thorough literature review conducted by Von Lampe (2012), research on organized crime usually falls into one of the following categories: a specific type of crime (Ohlemacher 1998; Suendorf 2001; Boberg 2018; Von Lampe 2003; Herz 2005; Sapelza 2017; Bulanova-Hristova and Kasper 2019) or crime group (Sergi and Lavorgna 2016), the structure of organized crime groups (Weschke and Heine-Heiß 1990; Mastrobuoni and Patacchini 2012), the logistics behind their illegal business dealings (Sieber and Bögel, 1993; Bögel 1994), or organized crime in a specific country and/or geographical region (Cheloukhine 2008; Spapens and Moors 2020; Kerner 1973; Kinzig 2004; Sciarrone and Storti 2014) (Von Lampe 2012). To date, the overwhelming majority of empirical research data on organized crime in the German context dates back to the late twentieth century and the early 2000s and is based on either interviews with or surveys of law enforcement personnel (Kerner 1973; Rebscher and Vahlenkamp 1988; Dörmann et al 1990; Sieber and Bögel 1993; Bögel 1994; Pütter 1998) or the analysis of court files – sometimes in combination with interviews (Weschke and Heine-Heiß 1990; Weigand and Büchler 2002; Kinzig 2004).

In the 1970s, Kerner (1973) was the first to conduct major empirical research on organized crime in Germany finding a growing internationalization of organized crime with a tendency towards the blurring of lines between ‘common’ crime, corporate crime and organized crime (Kerner 1973: 294). In the following years a number of research projects on organized crime in Germany with different foci were undertaken (Rebscher and Vahlenkamp 1988; Dörmann et al 1990; Weschke and Heine-Heiß 1990; Sieber and Bögel 1993; Bögel 1994). Based on these research projects, three central aspects of organized crime in Germany became apparent: First, the structure of organized crime groups tends to be more loose and network-oriented than strictly hierarchical (Rebscher and Vahlenkamp 1988: 181f.; Weschke and Heine-Heiß 1990: 44ff.). Second, violence and isolation from outside influences are not as integral to organized crime groups as previously assumed (Rebscher and Vahlenkamp 1988: 184ff.). Third, the pursuit of the biggest financial gain is the guiding principle of organized crime which leads to the use of business-like logistics in their illegal dealings (Rebscher and Vahlenkamp 1988: 183; Sieber and Bögel 1993; Bögel 1994). Kinzig (2004) attested a certain degree of ‘professionalism’ and conspiracy to organized crime groups. With his extensive research work over the last decades, Klaus von Lampe’s perspective on organized crime is central to the understanding of the phenomenon in the German context and his research is also among the most cited works on organized crime in Germany. Von Lampe’s research primarily focuses on the illegal cigarette trade in Germany (2002, 2003, 2005, 2006, 2015) – and beyond (Von Lampe and Kurti 2016; Von Lampe et al. 2016). In fact, according to von Lampe, the illegal cigarette black market is one particular area in which “large, structurally differentiated illegal enterprises” are somewhat destined to emerge due to the sheer number of contraband cigarettes being produced and sold which then may lead to an “increased integration [of their illegal business] into the legal economy” (Von Lampe 2015: 308f.). Additionally, Von Lampe also extensively worked and continues to work on conceptual questions (2001, 2008, 2013, 2019) as well as on questions of how to assess organized crime (2005). Aside from that, in the last few years a few smaller studies and dissertations on specific types of organized crime, e.g. human trafficking (Herz 2005; Sapelza 2017) or money laundering (Boberg 2018), have been undertaken. Still, large-scale nationwide empirical research projects as to the overall extent, the quality and/or the status quo of the general phenomenon of organized crime in Germany have been blatantly absent over the last 15 + years.

In contrast, European criminology is continuously contributing to the scholarly knowledge on organized crime. While several researchers focus on the (non-)existence of specific organized crime groups in given European countries (Sciarrone and Storti 2014; Blok 2008; Siegel and Turlubekova 2019), other researchers examine specific types of crimes in connection with organized crime groups. For example, Paoli (2003) compared organized crime groups’ activities in the illegal drugs trade in a trinational study of Germany, Italy and Russia (Paoli 2003). Siegel (2008) conducted research on the Antwerp diamond market and organized crime groups. Bezlov and Gounev (2008) researched organized crime and car thefts in Bulgaria and found that the “old hierarchical mafia-like model has disappeared” in favor of criminal network structures (Bezlov and Gounev 2008: 77). In 2015, the European-wide comparative research project ARIEL (“Assessing the Risk of the Infiltration in EU MSs Legitimate Economies”) found different levels of infiltration of legal businesses across Europe. The different levels ranged from a “strong penetration of the economic and political spheres” in Southern Italy (Savona and Berlusconi 2015: 44) to countries such as Sweden or the Netherlands where it is not (yet) as big a problem.

In light of the current COVID-19 pandemic a small number of criminological research has already been conducted on the influence of COVID-19 on organized crime. In that context, Zivotic and Trajkovski (2020) describe organized crime as a “living organism” that “avoids disappearance and fights for survival trying to adapt to the new situation” (Zivotic and Trajkovski 2020: 1003). Thereby, Zivotic and Trajkovski highlight two ‘superpowers’ of organized crime groups: their flexibility and adaptability. In fact, organized crime groups quickly discovered new lucrative businesses, started counterfeiting and selling products that where highly in demand, e.g. masks, disinfectant or (negative) COVID-19 tests, created innovative internet scams, e.g. COVID-19-related phishing schemes, and attempted to gain possession of the limited COVID-19 vaccines (Zivotic and Trajkovski 2020; Schotte and Abdalla 2022). Regarding said potentially severe threat of organized crime groups’ activity targeting COVID-19 vaccines around the world, Interpol issued an “Orange Notice”^2^ in December of 2020 in order to warn law enforcement agencies in all 194 member countries of this threat (Interpol 2020). According to a United Nations Office on Drugs and Crime (UNODC)’s (2020) research brief, the pandemic presented both opportunities and challenges for organized crime groups. On the one hand the crisis provided an opportunity for organized crime groups who had previously already infiltrated legitimate businesses to further strengthen their influence and widen their territory (UNODC 2020: 29). On the other hand, organized crime groups, too, were struggling with lockdown measures, the closing of borders, travel restrictions and curfews which, at least momentarily, disrupted their criminal activities (UNODC 2020: 29ff.). However, A COVID-19-centered report of the European Monitoring Centre for Drugs and Drug Addiction (EMCDDA) (Cunningham et al 2021) found that organized crime groups in the Western Balkans were rather “resilient to disruption to their business models” in light of COVID-19-related restrictions (Cunningham et al 2021: 2): While some modi operandi had to be adapted to COVID-19 circumstances, no significant disruptions or lasting changes occurred in terms of the illicit drug markets (Cunningham et al 2021: 3). Similarly, increased activities of organized crime groups were reported in Latin America (La Balmori de Miyar et al. 2021). In addition to continuing their illegal business dealings by adapting to COVID-19 circumstances, organized crime groups displayed their power by taking over quasi-governmental functions as their interests in the fight against the pandemic were quite closely aligned with those of the legitimate governments. Particularly in some South American countries where the legitimate governments are perceived as rather ‘weak’, organized crime groups used their quasi-governmental position to strictly enforce COVID-19 measures e.g. social distancing, quarantine or curfews (Aziani et al 2021; Moncada and Franco 2021; Fajardo 2020) or provide food packages (Felbab-Brown 2020) in certain neighborhoods.

With respect to Germany, Dellasega and Vorrath (2020) found that organized crime groups, particularly in the beginning of the pandemic, did not only adapt their business to include the sale of inadequate or non-existing medical supplies, e.g. masks, to desperate hospitals and politicians. They also profited from the widespread fear of a collapsing economy which was to be prevented through unbureaucratic state grants for all businesses struggling due to state-imposed COVID-19 measures. Naturally, organized crime groups exploited this opportunity and applied for numerous grants for non-existing businesses (Dellasega and Vorrath 2020). Furthermore, it was predicted that struggling legitimate businesses, e.g. restaurants, may become a prime target for the infiltration of organized crime groups in pandemic times (Dellasega and Vorrath 2020). Namli (2021) studied street drug trafficking and drug prices during and after the ‘first wave’ of COVID-19 in Germany and found no significant changes in terms of drug distribution – aside from nationally imposed curfews at times limiting drug sales at night.

In addition to influencing organized crime the pandemic naturally also influenced the work of law enforcement agencies all over the world. Already in April of 2020, Europol presented a first report on the way in which the pandemic will and/or could potentially influence serious and organized crime as well as the work of law enforcement in Europe based on data from all member states (Europol 2020). According to this report, law enforcement authorities – just as organized crime groups – were forced to “quickly adapt to the changing circumstances” which not only led to a sudden increase in the use of online tools and digital technology but also to an “unprecedented number of law enforcement officers working remotely” (Europol 2020: 15). Additionally, in a comparative study of 27 countries Maskály et al. (2021) found that COVID-19 measures also led to a decrease of in-person police training, of in-person responses to calls for service and in some instances to a decrease or at least delay in arrest practices (Maskály et al 2021: 280 f.) Considering that the work of law enforcement agencies is at least in part rather “hands-on”, the pandemic presented major challenges to law enforcement agencies worldwide.

Hence, while some research on organized crime and COVID-19 has been conducted, so far, empirical research on the pandemic influences of law enforcement agencies’ investigations of organized crime in Germany is still lacking. Following the presentation of our theoretical and methodological approach to this study, we set out to fill the void regarding the narratives provided by law enforcement personnel in terms of the influence of COVID-19 on their work and we present central findings from our interview study with law enforcement personnel.

## Methodological approach and interview study

### Situational Analysis and Grounded Theory as a framework

In terms of methodological procedure, we have chosen an approach which corresponds well to a large-scale explorative study: Adele Clarke’s Situational Analysis. Clarke’s postmodern Situational Analysis is deeply rooted in Symbolic Interactionism and Foucault’s Discourse Analysis and in this approach the research situation itself becomes the ultimate object of research. The methodological marriage of Symbolic Interactionism and Foucault’s Discourse Analysis entails a qualitative and reflexive approach, which is led by the techniques of Glaser and Strauss’ (1967) Grounded Theory Methodology. While Grounded Theory Methodology has focused on generating “basic social processes” occurring in the data, Situational Analysis’ fundamental assumption is that everything in a given situation both constitutes and affects everything else in that situation (Clarke et al. 2018: 4 ff.). Clarke explains her Situational Analysis as a postmodern extension of Grounded Theory Methodology: It is not about uncovering the one basic social process, as in Grounded Theory, but about understanding which elements are contained in a phenomena-based research situation and how they relate to each other. Hence, the complexity of the research situation itself is to be made the object of analysis. Furthermore, it is important to adopt a reflexive research attitude that considers one's own involvement in the object of investigation. The criticism of Strauss and Glaser's Grounded Theory Methodology is that the researcher remains invisible in the analysis since “[t]his constitutes a multifaceted denial that we are, through the very act of research itself, directly in the situation we are studying” (Clarke et al. 2018: 34 f.) In Situational Analysis, this is taken up analytically and transformed into a self-reflexive approach that considers the positionality and knowledge of the researcher in the situation being researched. In this article, we aim to provide an example of how one's positionality can be incorporated into answering the research question without mirroring oneself or providing a confessional script (Schmidt 2022). In addition, central discourses and bodies of knowledge of the research situation also become the object of analysis.

All in all, Situational Analysis encourages an open, inductive approach and helps gather the characteristics of the research situation in its entirety. As an analytical heuristic, Clarke, therefore, proposes a mapping strategy consisting of “situational maps”, “social world/arena maps” and “positional maps” with every type of map serving a different function in the analysis. As situational maps help clarify and visualize human, non-human, discursive and other elements important for the respective research situation (Clarke et al. 2018: 127 ff.), a situational map was created and used to ensure that all elements of the entire research situation in question – researching organized crime in times of COVID-19 – were considered and analyzed.

### Who, why and how? – selection of interviewees

At the core of our above-mentioned project – the empirical exploration of organized crime in Germany – is a large nationwide qualitative interview study with both professionals involved in the fight against organized crime and persons who are currently imprisoned due to their supposed affiliations with organized crime groups. This paper, however, is only based on our nationwide interview study in which 32 semi-structured interviews with interviewees from the police, the customs criminal investigation office, and public prosecutors' offices which were all conducted in the Fall and Winter of 2021. Prospective interviewees were generated through the help of associated law enforcement agencies and subsequently contacted with an official invitation to participate in our nationwide interview study. While getting access to the research field in search of prospective gatekeepers and potential interviewees for qualitative interview studies often proves to be one of the first pitfalls in criminological research (Fitz-Gibbon 2016), our fear in this context was thoroughly unfounded. Our calls for participation in our interview study were met with an abundance of ‘experts’ from law enforcement agencies expressing their willingness and eagerness to participate and share their professional knowledge on organized crime in Germany. The interviewees were chosen based on their professional expertise and daily experience in combatting organized crime in Germany. All interviews were conducted in German^3^ by the two authors of this paper and the duration of the interviews ranged between 40 min and 3 h with most of the interviews taking around 90 min. Of the 32 interviews, 19 interviews were conducted as in-person face-to-face interviews, 8 interviews were conducted by phone and 5 interviews via video.

In line with our qualitative methodological approach, the interview questions were designed to generate narrative and open-ended replies. This was done to gain insights into the interviewees’ professional knowledge, their patterns of interpretation regarding the phenomenon as well as the institutional handling of organized crime investigations. In doing so, we followed Meuser and Nagel’s (2009) methodological guidelines for so-called ‘expert interviews’. In our study, we classify experts as professionals who are “holding the power of articulation” (Elias and Scotson 1994) and who have specific knowledge about organized crime due to their profession (Petintseva et al. 2020).

### Qualitative methods and analysis in practice during the pandemic

**Fig. 1:**
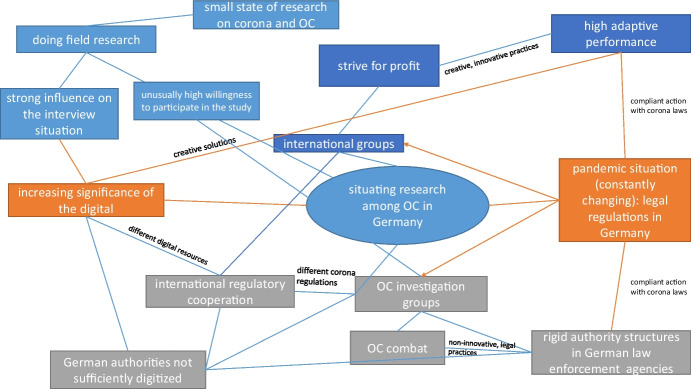
Own presentation

The idea for this article stems directly from our interviews as the CODIV-19 pandemic was made a topic by our interviewees in the context of their work in investigating organized crime. Ensuring informed consent of all interviewees, the interviews were recorded and later fully transcribed. All interview excerpts in which COVID-19 was mentioned were collected and analyzed in terms of narratives provided by the interviewees on how the pandemic influenced organized crime and their work in its combat. In a joint analysis group, all interview sequences were analyzed using Grounded Theory Methodology: To begin with, the interview excerpts were open-coded in order to break up the data and then axial coding was employed to revise, discard, rename, or refine previously found categories. In a third analytical step, an (ordered) situational map was created in order to understand the categories as elements of the research situation and to visualize all the elements relevant to both the interviewees’, our observations and the way in which they relate to each other. This approach was deliberately taken as it allows for the exploration of a rarely studied phenomenon to be understood in its entirety and the way in which it is constructed by actors in the field. In fact, such mapping strategies are often used to visualize analysis processes and do not claim to be complete (Clarke et al. 2018: 127 f.). That said, following the presentation of our empirical findings, we present an ordered situational map (Fig. 1) by way of visualization which takes up both the pandemic as a condition for the research, and the presentation of the interviewees’ central narratives regarding COVID-19 and organized crime. In doing so, we also take into consideration recent work on narrative criminology which understands narratives not only as a depiction of social reality but also as a way to produce said reality (Presser and Sandberg 2019). This goes hand in hand with the symbolic-interactionist assumption of the Thomas Theorem which states that “if men define situations as real, they are real in their consequences” (Thomas and Thomas 1928).

In addition to presenting central findings from the interview study, we also want to take the opportunity to reflect on the influence of COVID-19 on the implementation of our interview study. In that sense and in line with Clarke’s Situational Analysis, we as researchers also become an analytical part of the research situation in question. Therefore, it is not only interesting how COVID-19 affects the fight against organized crime in Germany, but it is equally interesting how the pandemic and precautionary measures affected our interview study. Hence, the objective behind this article is twofold: It is intended to be both an overview of our findings and a reflection on the methodical conduct of our study.

## Empirical findings

As the interviews were conducted in the midst of a pandemic, the interviewees themselves raised the issue of the influence of COVID-19 on organized crime and on their work in the fight against it. While the extent of the narratives in this regard varied in depth and length over all the interviews, three central narratives could be identified: Us vs. Them, Nationalization vs. Internationalization, and Conservatism vs. Innovation. In the following, we will present these central narratives by way of providing answers to our research question: Which narratives do law enforcement agencies provide for the impact of COVID-19 on organized crime and their work in the investigation thereof?

### Narrative: “us” vs. “them”

The corona discourse and its influence on the work of German law enforcement agencies with respect to organized crime was introduced by one interviewee as follows:

“Well, they had the same problems in terms of corona. But aside from that, I’d rather say that we were at a disadvantage.” (Interview 1)

Within this quote, the interviewee creates a strict dichotomy of ‘us’ – law enforcement agencies – versus ‘them’ – organized crime groups – which, although always present in their respective line of work, appears to become even more pronounced in pandemic times. By speaking about the dichotomy of ‘we’ versus ‘they’ in lieu of ‘they’ versus ‘I’, the use of the plural also clearly marks the element of a united law enforcement agency ‘front’ against organized crime groups. Yet, even though the pandemic with all its restrictions and societal changes equally influenced everyone living in a certain geographical region and created the same social reality for everyone, law enforcement agencies – at least according to the interviewee – were still at a disadvantage when compared to organized crime groups. In this construction of German law enforcement agencies as the disadvantaged and/or disproportionately burdened by the pandemic and organized crime groups as the fortunate ones being able to better deal with the same COVID-19-related challenges, certain traces of both envy and admiration for organized crime’s ability to regroup in a crisis can be detected.

The presented juxtaposition – the “us vs. them” – is a common thread in the way in which law enforcement agencies and so-called criminals of any sorts are constructed. In fact, the “us vs. them” is the necessary prerequisite for the work of law enforcement agencies, as their entire existence as well as all of their investigative work depends on the construction of the ‘criminals’ with their illegitimate actions as being directly opposed to the crime-fighting, safety-granting law enforcement personnel. When it comes to organized crime, the respective other is to some extent even more constructed as the complete opposite of law enforcement. Said underlying opposition or competition of some sorts leads to organized crime groups being imagined as something mystical, or even ghost-like, that law enforcement agencies cannot quite grasp and only rarely catch. According to all the interviewees, this constant state of being at least one or two steps behind the opposed criminals is something inherently connected to the phenomenon of organized crime and as such both a perfect illustration and manifestation of the well-known ‘cops and robbers’-narrative in modern times. In that sense then, it becomes clear that COVID-19 had differential effects on law enforcement agencies and organized crime groups and further strengthened the concept of an “us” vs. “them”. That said, the following analysis of the narratives, “nationalization vs. internationalization” and “conservatism vs. innovation”, will provide further empirical insights into other narratives provided by law enforcement agencies for such differential effects of COVID-19 under the same conditions on both sides of this dichotomy.

### Narrative: nationalization vs. internationalization

In line with the state of research on organized crime, the interviewees presented organized crime as an international phenomenon:

“Organized crime is certainly not stopped by national or state borders (…).” (Interview 2)

This ability of organized crime groups to network across borders – especially when faced with the COVID-19 pandemic – was often problematized by our interviewees as the pandemic drastically amplified this general challenge of German law enforcement agencies. The strict legal separation of the various German law enforcement agencies combined with the federal system in Germany created a large variety of – sometimes opposing – measures to combat the pandemic. But not only within Germany, but also internationally the different law enforcement agencies struggled with such differing COVID-19 control strategies. For example, one interviewee described how they were to conduct undercover investigations together with colleagues of another country. While the German officers were required to always wear masks, masks were not mandatory for their foreign colleagues, which according to the interviewee, made it quite obvious that he and his colleagues were Germans.

Although interviewees reported that international cooperation in and across Europe has improved in recent years – especially through institutional cooperation initiatives and networks, e.g. Europol and Eurojust, the COVID-19 pandemic triggered a new focus on the national. Despite all differences in COVID-19 regulations, however, Europe was united by one central measure: the implementation of contact restrictions better known as social distancing. Problems that already existed before the pandemic, e.g. the use of different software programs in the individual federal states in Germany, were further exaggerated by the sudden increased reliance on digital exchange (see 4.3 conservatism vs. innovation). As at some point most countries resorted to closing their borders, the interviewees reported that this also presented challenges for organized crime groups to pursue their illegal businesses.

“In part, it became more difficult for our perpetrators. That’s what we now see in the analyses of Encro and Sky. They had problems to get their stuff here when the borders were closed. But there was still enough here. No one was screaming ‘Help, I am out of stuff’. It just became more expensive” (Interview 10)

This interview sequence illustrates, that while the COVID-19 measures are seen as problematic for organized crime groups’ business, they are not perceived as having put an end to criminal activity. Instead, as the interviewee describes, products have become more expensive. It thus appears that even during the pandemic the (illegal) market was regulated by demand as the interviewees attest an extreme adaptive capacity to the organized crime groups, which became particularly evident during pandemic containment measures (see 4.3 Conservatism vs. Innovation).

This (international) business acumen of organized crime leads to an interesting observation made by various interviewees. According to their reports, organized crime groups were said to have been exceptionally compliant with COVID-19 regulations, particularly at the borders. It thus becomes clear that, just as the interview sequence in the introductory passage of the empirical findings section illustrated, the internationalization of the fight against organized crime in times of COVID-19 is also interpreted as a challenge, but only for the law enforcement agencies. This is mainly because perpetrators of organized crime are described as adaptive, profit-maximizing, and innovative (see 4.3 Conservatism vs. Innovation). While in terms of the international aspect of organized crime the pandemic presents a new opportunity for organized crime groups, it proves to be rather difficult for law enforcement agencies in Germany.

### Narrative: conservatism vs. innovation

As illustrated by the previous section, the COVID-19 pandemic with its continuously changing regulations presented a major challenge for both organized crime groups and law enforcement agencies. Going back to the idea that organized crime groups were at an advantage in the pandemic even when both German law enforcement agencies and organized crime groups were, in fact, faced with the same new conditions (see 4.1 Us vs Them), the way in which they both dealt with the situation was quite different: conservatism (law enforcement agencies) versus innovation (organized crime groups).

When it comes to organized crime and organized crime groups, it is often pointed out that it is generally not easy to classify organized crime as such due to definitional difficulties. Next to the extraordinarily strong strive for financial gain, it is said that one of the trademarks of organized crime groups is their ability to almost instantly adapt to new circumstances. According to our interviewees, traditionally, almost all organized crime groups in Germany are at least in some way involved in the trafficking and distribution of drugs, as this is, apparently, the area with the biggest profit margin. Further traditional activities include weapons trafficking, human trafficking and other cases of economic crime and fraud. Still, organized crime groups’ high adaptability to new and/or unknown situations and the innovative drive is what ultimately put(s) organized crime groups at an advantage during the pandemic: Organized crime groups put their innovative spirit to work and within only a couple of weeks of the pandemic they realized that there is a “niche” or vacuum that they could exploit. This was pointed out by one of the interviewees who said that “selling counterfeit face masks […], [COVID-19] tests or pocketing governmental corona grants and so on, is classic organized crime. They are right there and realize that there is a gateway for them to exploit” (Interview 5). Here, the interviewee once again highlights the adaptability and sense of creativity of organized crime groups which allows them to quickly discover new business avenues. In fact, the interviewee constructs this high adaptive power as the core trait of organized crime. Similarly, another interviewee stressed the high adaptive performance coupled with the strong drive for financial gain of organized crime groups in the pandemic by saying:

“Organized crime takes place where money can be earned and the COVID-19 pandemic, in that sense, opened up new business areas in which one could quickly and easily make a lot of money.” (Interview 3)

Later that same interviewee presented an example for said high adaptive performance by referring to one organized crime group who was previously active in the energy sector and due to the pandemic suddenly switched businesses and sold “millions of tons of disinfectant […] and face masks” which were either faulty or non-existent (Interview 3). To further illustrate the success of this particular organized crime groups’ change in businesses the interviewee mentioned that their profits in this regard were roughly two million euros. In the words of another interviewee such innovative power and creativity is innate to – at least successful – organized crime groups. According to him, it is especially the “well-organized/’good’ organized crime groups […] that have a super good feeling for trends” (Interview 11). Said good intuition coupled with a certain knack for business allows organized crime groups to make the most of a given situation and to “unbelievably quickly adapt to new opportunities” (Interview 11). Another example of said adaptability to COVID-19 circumstances was introduced by another interviewee who explained that organized crime groups adjusted their pre-existing modus operandi to the pandemic and created new COVID-19-inspired variations of common modi operandi. In line with the introductory quote, the ‘respect’ for the ‘enemy’ and a certain degree of silent admiration for the creativity and innovative spirit of organized crime groups surfaces throughout all the interviewees’ narratives.

While organized crime groups due to their trademark degree of creativity and adaptability were not stopped for too long due to COVID-19 related restrictions, the work of the German law enforcement agencies took a hard hit according to our interviewees. The relatively sudden and rather severe COVID-19 measures implemented by the German federal government in the spring of 2020 naturally also influenced the work of German law enforcement agencies. As German bureaucratic institutions – both locally and nationally – are well known for being rather inflexible and resistant to changes in all respects, it is not surprising that law enforcement agencies were strongly influenced by COVID-19. In the words of our interviewees, the sudden COVID-19 situation did “massively impact” (Interview 5) their work and presented a “humongous problem” (Interview 4) for the way that law enforcement agencies organize their investigations in teams. This serves to illustrate the fact that in the context of the investigative work the COVID-19 restrictions were exclusively constructed as problematic. Government ordered social distancing, directives to work from home, and advisories to minimize all non-imperative contact with other people also presented a challenge for our interviewees because even though “organized crime investigations are a team effort” (Interview 4), investigation teams were not allowed to work together at the office. In fact, law enforcement agency personnel had to divide themselves into ‘subteams’ due to lack of physical office space which heavily impacted the way in which information was circulated within their teams as regular face-to-face team meetings were no longer allowed. That said, their investigative work was heavily impacted for almost an entire year with the situation only improving once law enforcement agency personnel were eligible to receive their COVID-19 vaccinations. This inflexibility and resistance of German authorities towards change is a side effect of the overall rather rigid and conservative bureaucratic structures which allow next to no room for quick reforms or adaptation to new situations such as the pandemic. Thus, this grants German law enforcement agencies few opportunities to measure up against the creativity, high adaptive performance and innovative spirit of their ‘enemies’: organized crime groups. Interestingly, said conservatism in German law enforcement was only actively discussed by one interviewee from a custom’s criminal investigation office who said that.

“We came up with completely new ideas which will further manifest itself in the work of the customs agencies even after the pandemic state of emergency. […] but by now, we have well-worn ways and means which allow us to almost work in the way we did before the pandemic.” (Interview 14)

By stipulating that he and his colleagues were, both struggling with the sudden changes and circumstances presented by the pandemic and using this experience to come up with solutions on how to deal with the situation at hand, a certain agency is manifested. Still, while he and his colleagues developed some new and innovative ideas on how to potentially improve their investigations and workflows which could possibly have a lasting positive impact for post-covid investigative work, he instantly refutes this capacity for innovation. He does so by referring back to their “well-worn” and proven strategies, which he presents as the ultimate work standard – a standard that needed to be reinstated as soon as the pandemic situation allowed.

Additionally, the COVID-19 pandemic served as a catalyst in terms of all things digital. Even though a lot of major technological advancements have already been introduced within the last couple of decades, making it hard to envision a world without the seemingly unlimited possibilities of the internet, the COVID-19 pandemic not only further accelerated its importance in today’s society but also served to make Germany painfully aware of the fact that digitalization in many places is more pipe dream than reality. That said, complaints about the technical equipment were a common narrative to be found in all our interviews with law enforcement personnel. While this is certainly not a new realization, it became a much more pressing issue due to COVID-19 and the way in which related measures such as social distancing led to the rapid implementation of video calls as primary means of communication in both work and leisure. This increased dependency on technology coupled with the extreme lack of appropriate technological equipment severely disrupted well-worn and established workflows in investigations of cases of organized crime. When German politicians demanded that all companies and authorities order most employees to – wherever possible – work from home in order to prevent the spread of the disease, this order naturally also applied to German law enforcement agencies. According to one interviewee, this presented a huge problem for their line of work because “just like people working in the free market economy, a lot of us started to work from home where we tried to somehow continue our work with the little technical equipment we had” (Interview 5).

One of the biggest hindrances to working from home in this context was rooted in the fact that the routinely conducted surveilling of all telecommunications of persons of interest within organized crime investigations must only be analyzed at the office. According to one interviewee, this “made it really difficult when it was not allowed to be at the office” (Interview 5) as the COVID-19 restrictions in this instance put a major toll on the quality of their investigations. While the surveillance of telecommunications still belongs within the standard repertoire of law enforcement and is routinely conducted in all major investigations, it continuously loses its prior power. Successful organized crime groups pride themselves on being well-versed in police tactics. That said, most members of organized crime groups are aware of the potential threat and risk of surveillance of their telecommunications. When up until a couple of years ago organized crime groups used to communicate rather openly on either landlines or cellphones, they have long since moved the majority of their illegal business dealings to encrypted messenger apps, e.g. WhatsApp, Threema, Signal, and most recently to entirely encrypted phone systems, e.g. EncroChat. Such onboard encryption of messenger apps and encrypted phones is basically impenetrable by anyone – even by law enforcement agencies – this technological advancement is purposefully used by organized crime groups to show the law enforcement agencies up. Hence, organized crime’s use of such innovative technology also serves to, once again, hold up the mirror to ‘old-school’ law enforcement personnel who still focuses on twentieth century technology, when organized crime groups have long since moved on and fully embraced the technological opportunities of the twenty-first century.

Considering all this, it is not really surprising that various interviewees said that they felt that they were at a disadvantage during the – at least first year of the – pandemic as compared to organized crime groups who they presented as having much more easily adapted to the new COVID-19 status quo.

### Situational map of combating organized crime during the pandemic

Following the presentation of the central findings from our interviews with law enforcement personnel, we now want to utilize a situational map (see Fig. 1) to illustrate the way in which all elements of the research situation in question – the influence of COVID-19 on the investigation of organized crime and qualitative research – are interdependent. Therefore, the situational map not only entails the narratives of the interviewees (dark blue and grey)^4^ but also the situation for us as researchers (light blue). For instance, the situational map visualizes the significant increase in the importance of the digital for both academia and law enforcement agencies and the way in which this influences interpretations of organized crime trends. We interpret the interviewees’ high willingness to participate in the study as a sign of the pent-up demand for German law enforcement personnel to talk about their needs when it comes to fighting organized crime in Germany. Said needs were apparently further exacerbated by the COVID-19 pandemic. The rigid structures of German law enforcement agencies in combination with constantly changing COVID-19 regulations, both at home and abroad, prevent the effective utilization of previously established means of international cooperation. The interviewees’ emphasis on the difficulties of the COVID-19 situation on their investigative work is in stark contrast to the presentation of highly adaptive organized crime groups who still manage to profit off of the pandemic which once again stresses the “us vs. them” narrative. In this way, organized crime is constructed as a mysterious crime phenomenon where both the participants and structures are adaptive, always thinking one step ahead and driven by the unwavering and unstoppable strive for profit.

## Conclusion – what’s the takeaway from this study?

The COVID-19 pandemic has presented everyone with challenges that call for creative solutions. The methodological approach of a situational analysis shows how supposedly separate spheres, such as academia, crime and its control, can be understood as an interdependent web with all having to operate under the same conditions due to COVID-19.

We have shown that – with only a few exceptions – limited attention has been paid to major empirical research on the general phenomenon of organized crime in Germany over the past 15 years. International research on organized crime is usually limited to specific groups, specific offenses, or specific geographic regions. A qualitative-explorative approach is rarely taken. Recent research on organized crime and COVID-19 picks up on some aspects that were also made significant by our interviewees. In line with other research, we found both a curtailment of criminal activity due to the pandemic and the use of new opportunities and business avenues e.g. the exploitation of COVID-19 government aids or grants (Dellasega and Vorrath 2020; Zivotic and Trajkovski 2020). However, our findings go further by showing how German law enforcement agencies with their rigid federal structures were forced to act in a normative, conformist manner that leaves little room for creative solutions. It also appears to be the case that narratives in the organized crime response have not necessarily changed as a result of the pandemic. Instead, existing interpretations about problems in combating organized crime in Germany primarily have been further exacerbated due to COVID-19. This is in stark contrast to the interpretations of organized crime groups’ adaptability. In addition, we address the conduct of our own research in times of the pandemic in an attempt to strengthen a reflexive approach to criminology.

By applying Grounded Theory and Situational Analysis in our analysis of the interview data, we identified three different dimensions (“us vs. them”, “nationalization vs. internationalization” and “conservatism vs. innovation”) which intertwine and present themselves both as narratives being made significant by the interviewees and as a phenomenological condition and result of the pandemic situation. The interlocking of these dimensions was visualized in a situational map (Fig. 1) in order to illustrate the complexity of the social situation. According to the narratives provided by our interviewees, organized crime is an international phenomenon that can hardly be dealt with in a national or agency-separate manner. COVID-19-related contact restrictions and social distancing regulations have led to a shift of all (social) life towards the digital. German authorities have had to deal with massive technological problems as a result: In some cases, technical equipment was lacking, many things could not be done while working from home, and different digital communication channels of respective agencies were incompatible. Thus, when the interviewees speak of organized crime groups’ innovative action, their flexibility and adaptability – organized crime’s superpowers (Zivotic and Trajkovski 2020) –, the interviewees are presenting organized crime groups in terms of a juxtaposition to their own institutionally and pandemic constrained action: the afore-presented “us” versus “them” narrative. Therefore, narrative contrasts are drawn that translate the consolidated interpretive pattern of “cops and robbers” into the pandemic era. Along the same lines our interview findings additionally stress the fact that while the situation of COVID-19 itself was the same for both law enforcement agencies and organized crime groups, the way to deal with and adapt to the situation was, in fact, different – hence “same but different”.

In addition to the three dimensions, however, one central narrative stood out that seemed to hold across all interviews. Even though the phenomenon of organized crime often remains very vague and examples are only given upon specific request, everyone agreed that organized crime can be recognized by its ultimate orientation towards profit. Thus, a dominant interpretation prevails that constructs organized crime as an internationally operating, highly- adaptable, and economically driven form of crime. The narrative of profit-orientation is presented as a teleological action and can thus be interpreted as an explanatory interpretation in which the existence of organized crime is attributed to illegal-economic action. In future studies on organized crime, its connection with white-collar crime must be further examined to be able to not only determine its similarities and differences but also to develop a suitable and effective way for law enforcement agencies to deal with organized crime.

Following a reflexive research approach, we also had to take into consideration the way in which the pandemic situation influenced our qualitative research. Just as COVID-19 required a high level of adaptability from both law enforcement agencies and organized crime groups, conducting this interview study also presented us as qualitative researchers with before unknown challenges. While under ‘normal’ circumstances it is a given that qualitative researchers spare no effort when it comes to traveling to their respective interviewees to conduct face-to-face interviews – the gold standard of qualitative interview research (Self 2021) –, we were forced to reevaluate the way we could responsibly collect our interview data under the ever-changing decree of COVID-19. In line with Gruber et al. (2020), we chose a mixed-mode design in order to be adaptive to the situation and left the mode of interviewing (face-to-face, phone, video) up to the interviewees. In doing so, we made sure that all our interviewees were free to decide which COVID-19-related risk they and their respective agencies were willing to take at that time. Most of the interviews could, in fact, be conducted face-to-face (19 out of 32 interviews), while only a few interviews were conducted by phone (8 out of 32 interviews) or via video (5 out of 32 interviews). In addition to influencing the organization of our interview study, the interview situations themselves were also marked by COVID-19-related regulations (e.g. face masks), so that the ongoing pandemic, in some way or other, always played a part in the interviews and the interview situation itself. Having said that, in our reflexive research approach, we therefore included the pandemic situation as an underlying current in our data analysis as – just as Clarke’s Situational Analysis proclaims – the research object in question and the research situation itself are always interrelated.

## Footnotes

1 The Research Alliance „Organized Crime 3.0 “ consists of 5 universities and research institutions throughout Germany and it is supported by various law enforcement agencies.

2 INTERPOL issues a number of different color-coded notices to police in tis member countries to warn and/or inform about international requests for cooperation or crime-related alerts. The so-called “Orange Notice” is issued “to warn of an event, a person, an object or a process representing a serious and imminent threat to public safety” (Interpol Fact Sheet; Interpol 2022)

3 For the purpose of this paper, the authors translated all interview excerpts into English as literally as possible.

4 For the colored figure, please refer to the open access version of this article.
